# Antibiotic Treatment of Suspected and Confirmed Neonatal Sepsis Within 28 Days of Birth: A Retrospective Analysis

**DOI:** 10.3389/fphar.2019.01191

**Published:** 2019-10-15

**Authors:** Jadon S. Wagstaff, Robert J. Durrant, Michael G. Newman, Rachael Eason, Robert M. Ward, Catherine M. T. Sherwin, Elena Y. Enioutina

**Affiliations:** ^1^Division of Clinical Pharmacology, Department of Pediatrics, University of Utah School of Medicine, Salt Lake City, UT, United States; ^2^Division of Medical Laboratory Science, Department of Pathology, School of Medicine, University of Utah, Salt Lake City, UT, United States; ^3^University of Utah Health Science Center, Salt Lake City, UT, United States; ^4^Department of Pediatrics, Wright State University, Boonshoft School of Medicine, Dayton Children’s Hospital, Dayton, OH, United States; ^5^Pharmaceutics & Pharmaceutical Chemistry, College of Pharmacy, University of Utah, Salt Lake City, UT, United States

**Keywords:** neonatal sepsis, antibiotic stewardship, rational prescribing, empiric therapy, newborns, bloodstream infections

## Abstract

Neonatal sepsis causes significant mortality and morbidity worldwide. Diagnosis is usually confirmed *via* blood culture results. Blood culture sepsis confirmation can take days and suffer from contamination and false negatives. Empiric therapy with antibiotics is common. This study aims to retrospectively describe and compare treatments of blood culture-confirmed and unconfirmed, but suspected, sepsis within the University of Utah Hospital system. Electronic health records were obtained from 1,248 neonates from January 1, 2006, to December 31, 2017. Sepsis was categorized into early-onset (≤3 days of birth, EOS) and late-onset (>3 and ≤28 days of birth, LOS) and categorized as culture-confirmed sepsis if a pathogen was cultured from the blood and unconfirmed if all blood cultures were negative with no potentially contaminated blood cultures. Of 1,010 neonates in the EOS cohort, 23 (2.3%) were culture-confirmed, most with *Escherichia coli* (42%). Treatment for unconfirmed EOS lasted an average of 6.1 days with primarily gentamicin and ampicillin while confirmed patients were treated for an average of 12.3 days with increased administration of cefotaxime. Of 311 neonates in the LOS cohort, 62 (20%) were culture-confirmed, most culturing coagulase negative staphylococci (46%). Treatment courses for unconfirmed LOS lasted an average of 7.8 days while confirmed patients were treated for an average of 11.4 days, these patients were primarily treated with vancomycin and gentamicin. The use of cefotaxime for unconfirmed EOS and LOS increased throughout the study period. Cefotaxime administration was associated with an increase in neonatal mortality, even when potential confounding factors were added to the logistic regression model (adjusted odds ratio 2.8, 95%CI [1.21, 6.88], p = 0.02). These results may not be generalized to all hospitals and the use of cefotaxime may be a surrogate for other factors. Given the low rate of blood culture positive diagnosis and the high exposure rate of empiric antibiotics, this patient population might benefit from improved diagnostics with reevaluation of antibiotic use guidelines.

## Introduction

Neonatal sepsis remains a significant cause of morbidity and mortality, particularly among preterm and low birth-weight neonates (Thaver and Zaidi, 2009; Shane et al., 2017). It is estimated that within 28 days of birth, 13% of neonatal mortality worldwide is caused by sepsis or meningitis (Liu et al., 2012). Neonatal sepsis is commonly divided into early-onset sepsis (EOS) and late-onset sepsis (LOS). EOS (≤3 days of birth) is generally associated with organisms transferred to the neonate from the mother while LOS (>3 days of birth) is usually associated with nosocomial or community acquired infections (Dong and Speer, 2015).

Diagnosing neonatal sepsis can be enigmatic—there is no consensus among clinicians or researchers. The current “gold standard” for diagnosis is a positive blood culture (Wynn et al., 2014). Blood culture-confirmed sepsis is only one facet of the disease; suspected sepsis in culture-negative cases is still considered clinical sepsis (Lukacs and Schrag, 2012; Wynn et al., 2014). Current recommendations call for empiric use of antibiotics before culture confirmation if sepsis is suspected, and possible continued antibiotic treatment if signs of sepsis persist when the culture is negative (Camacho-Gonzalez et al., 2013; Shane et al., 2017). Because of this approach, antibiotics are frequently administered in neonatal intensive care units (NICUs). In a study of 253,651 NICU neonatal records within the Pediatrix Medical Group database, the two most commonly administered drugs were ampicillin (69%) and gentamicin (58%); additionally, 18% of neonates were exposed to cefotaxime and 10% to vancomycin (Clark et al., 2006). With an increasing emphasis on antibiotic stewardship, it is essential to understand how neonatal sepsis is confirmed and treated, and to identify opportunities for reducing antibiotic use.

A retrospective analysis was conducted using data obtained from The University of Utah Health system, comprising four hospitals and 12 community clinics. Our objective was to thoroughly describe the blood culture confirmation and antibiotic treatment of neonatal sepsis within this hospital system. Antibiotics used to treat culture confirmed and unconfirmed sepsis are compared for both early and late onset sepsis over a 12-year period.

### Methods

Collection of medical records for analysis was approved by the University of Utah Institutional Review Board (IRB_00091312). Records were collected from neonates born and/or admitted to the University of Utah Hospitals within 28 days of birth between January 1, 2006, and December 31, 2017, with ICD9 billing code 771.81 or ICD10 code P36 (newborn sepsis). Neonates with major birth defects such as spina bifida, gastroschisis, congenital heart defects (excluding patent ductus arteriosus and patent foramen ovale), Down syndrome, encephalocele, and microcephaly or neonates born from mothers with a family history of congenital immune disorders (x-linked agammaglobulinemia, x-linked neutropenia/myelodysplasia, common variable immunodeficiency, severe combined immunodeficiency, severe congenital neutropenia, Shwachman-Diamond syndrome, and Kostmann syndrome) were excluded from data collection.

We categorized these patients as suspected early-onset sepsis (EOS) when any blood culture was drawn and antibiotics were initiated ≤3 days of birth and suspected late-onset sepsis (LOS) when any blood culture was drawn and antibiotics were administered >3 but ≤28 days of birth and ≥7 days after a positive blood culture associated with confirmed EOS. Neonates with suspected late or early-onset sepsis are not mutually exclusive, that is, a single neonate can be categorized as both suspected early and late-onset sepsis if it meets the criteria for both. Neonates suspected of having sepsis were further divided into two categories, confirmed sepsis and unconfirmed sepsis. These categories are mutually exclusive (i.e., a single neonate suspected of having LOS must be either confirmed or unconfirmed LOS).

Only blood cultures were used to confirm neonatal sepsis. Centers for Disease Control and Prevention (CDC) bloodstream infection criteria were adapted for use to confirm sepsis with some modifications (Horan et al., 2008). According to these guidelines, if there was a recognized pathogen in a blood culture, sepsis was confirmed. If a common skin contaminant was cultured, and the organism was cultured again in the following 48 h, sepsis was also confirmed. If a common skin contaminant was cultured, and the organism was not cultured again in the following 48 h, then these cultures were possibly contaminated and these patients were excluded from analysis as ambiguous cases. The following microorganisms were considered common skin contaminants: diphtheroids, Bacillus spp., Micrococcus spp., non-pathogenic Neisseria spp., gram-positive rods, coagulase negative staphylococci (CoNS), and non-pathogenic streptococcal species (e.g., Streptococcus viridans) (Hall and Lyman, 2006). We also analyzed cerebrospinal fluid (CSF) cultures.

Antibiotic exposure was measured by days of treatment (DOT) and length of treatment (LOT) (Ibrahim and Polk, 2014). DOT is measured by multiplying the number of doses by the dosing interval in days for each antibiotic administered. DOT is normalized to per 1,000 patient days (PD) which is the mean length of stay multiplied by 1,000. LOT is measured by the number of calendar days a patient received any antibiotic. LOT is normalized to per admission and per treatment course. LOT/course is equivalent to the mean number of days that neonates are treated for an episode of sepsis. Antibiotics administered topically were not included.

Treatment courses were calculated as continuous periods of antibiotic exposure with no gap in administration greater than or equal to two calendar days to accommodate for extended intervals of aminoglycoside dosing in extremely low birthweight newborns. In some cases, there was continuous administration of antibiotics from within three days of birth to well beyond three days after birth where there was an obvious switch from treatment of unconfirmed EOS to treatment of suspected LOS. For example, in one neonate, a negative culture was drawn on day one of life and ampicillin and gentamicin were administered as treatment for EOS. At day five of life, more cultures were drawn, ampicillin administration ceased, and vancomycin administration began as treatment for LOS. In these cases, we considered treatment courses ending for EOS and treatment courses beginning for LOS when there was a blood culture drawn after three days of birth, then a change in antibiotic regimen on or after the same calendar day.

Severity of illness and risk of mortality were measured using All Patient Refined Diagnosis Related Groups (APR-DRG). Univariate comparisons were evaluated using Student’s *t*-test for continuous variables and chi-squared test or Fisher’s exact test for categorical. Median values are reported with an interquartile range (IQR) in brackets and mean values are reported with standard deviation (SD) in parenthesis. Trends over time were analyzed using simple linear regression for continuous variables, logistic regression for dichotomous variables, or one-way ANOVA for severity of illness and risk of mortality. Statistical tests were considered significant when p < 0.05. All analyses were undertaken with R version 3.5.3 (R Core Team, 2019) in conjunction with the dplyr version 0.8.0 R package.

## Results

**Figure 1:**
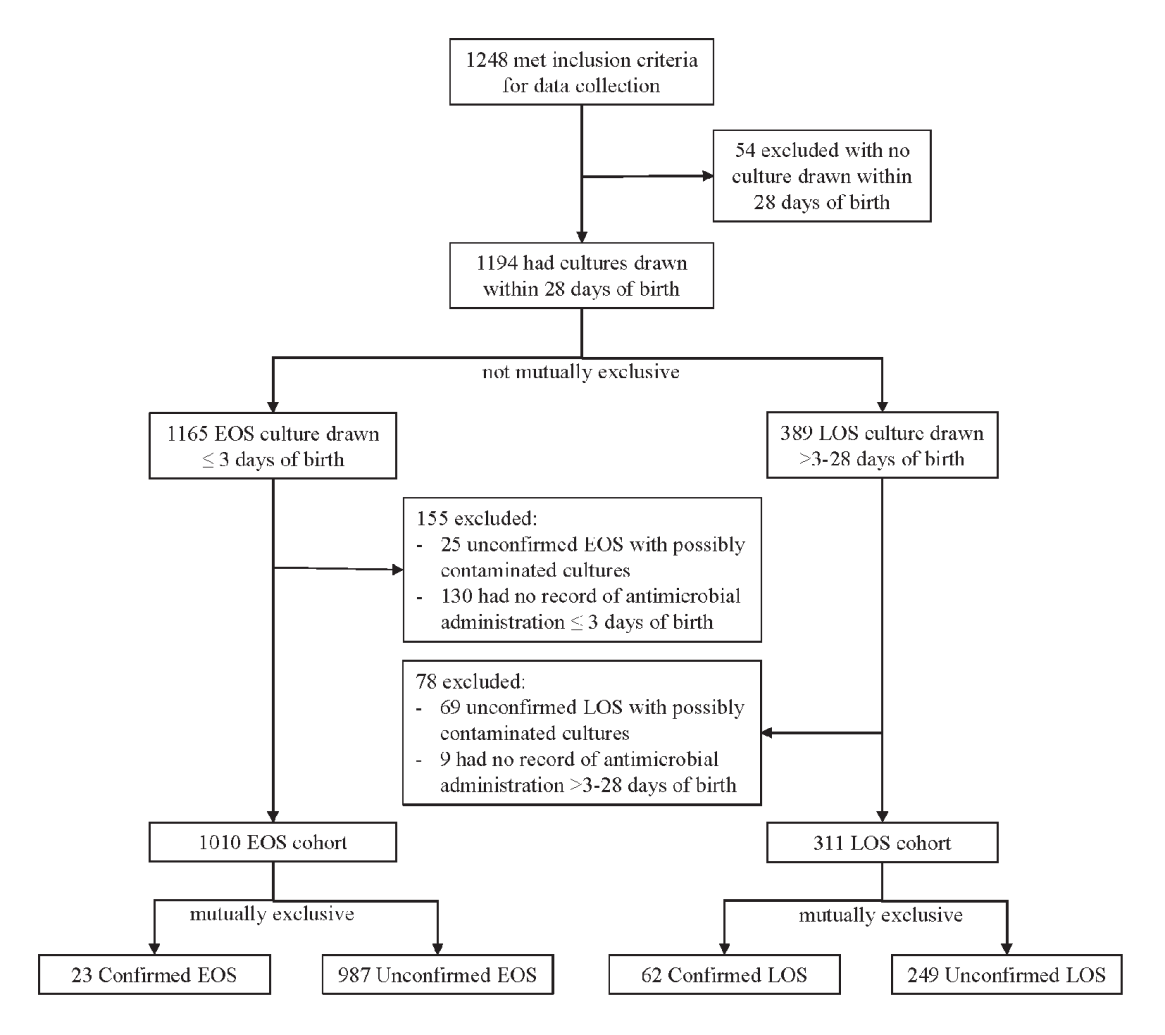
Flow chart of study cohort selection.

A total of 1,248 neonates met inclusion criteria for data collection, 1,010 met inclusion criteria for the EOS cohorts and 311 met inclusion criteria for the LOS cohorts (**Figure 1**). Patient demographics, mortality rate, length of stay, severity of illness, risk of mortality, birth weight, and gestational age for each cohort are compared in **Table 1**. Confirmed cases of LOS were more likely to include neonates with lower gestational age, lower birth weight, and higher severity of illness than neonates in unconfirmed cases of LOS (p < 0.01).

**Table 1:** Cohort demographics.

	Early-Onset			Late-Onset		
	Unconfirmed	Confirmed	P value	Unconfirmed	Confirmed	P value
**Number of cases**	987	23		249	62	
**Mortality within 35 Days of Birth (%)**	26 (2.6)	2 (8.7)	0.27	10 (4.0)	6 (9.7)	0.14
**Length of Stay in Days (median [IQR])**	23 [8, 57]	41 [15, 57]	0.50	58 [34, 101]	69 [23, 101]	0.72
**Risk of Mortality (%)**						
Extreme	118 (12.0)	2 (8.7)	0.46	50 (20.1)	14 (22.6)	0.46
Major	375 (38.0)	13 (56.5)		134 (53.8)	36 (58.1)	
Moderate	231 (23.4)	5 (21.7)		38 (15.3)	7 (11.3)	
Minor	255 (25.8)	3 (13.0)		24 (9.6)	3 (4.8)	
Missing	8 (0.8)	0 (0.0)		3 (1.2)	2 (3.2)	
**Severity of Illness (%)**						
Extreme	319 (32.3)	11 (47.8)	0.42	134 (53.8)	45 (72.6)	0.04
Major	342 (34.7)	8 (34.8)		88 (35.3)	12 (19.4)	
Moderate	223 (22.6)	2 (8.7)		14 (5.6)	2 (3.2)	
Minor	95 (9.6)	2(8.7)		10 (4.0)	1 (1.6)	
Missing	8 (0.8)	0 (0.0)		3 (1.2)	2 (3.2)	
**Patients with other Positive Cultures within 28 Days of Birth**						
Lungs/Airway (%)	75 (7.6)	2 (8.7)	0.12	40 (16.1)	18 (29.0)	0.57
Urine (%)	34 (3.4)	0 (0.0)		25 (10.0)	6 (9.7)	
Cerebrospinal Fluid (%)	3 (0.3)	1 (4.3)		4 (1.6)	1 (1.6)	
**Gestational Age (%)**						
>= 37 weeks (term)	272 (27.6)	4 (17.4)	0.68	14 (5.6)	1 (1.6)	<0.01
32–37 weeks (preterm)	281 (28.5)	7 (30.4)		46 (18.5)	4 (6.5)	
28–32 weeks (very preterm)	212 (21.5)	5 (21.7)		75 (30.1)	15 (24.2)	
<28 weeks (extreme preterm)	222 (22.5)	7 (30.4)		114 (45.8)	42 (67.7)	
**Birth Weight (%)**						
>= 2.5 kg (normal)	332 (33.6)	4 (17.4)	0.18	19 (7.6)	2 (3.2)	<0.01
1.5–2.5 kg (low)	275 (27.9)	6 (26.1)		52 (20.9)	6 (9.7)	
1-1.5 kg (very low)	156 (15.8)	7 (30.4)		61 (24.5)	9 (14.5)	
<1 kg (extremely low)	224 (22.7)	6 (26.1)		117 (47.0)	45 (72.6)	
**Male Gender (%)**	535 (54.2)	14 (60.9)	0.81	118 (47.4)	33 (53.2)	0.50
**Race (%)**						
White or Caucasian	528 (53.5)	11 (47.8)	0.68	140 (56.2)	35 (56.5)	0.58
Asian	23 (2.3)	0 (0.0)		3 (1.2)	1 (1.6)	
Black or African American	26 (2.6)	0 (0.0)		8 (3.2)	0 (0.0)	
Pacific Islander	11 (1.1)	1 (4.3)		6 (2.4)	1 (1.6)	
American Indian	12 (1.2)	0 (0.0)		2 (0.8)	1 (1.6)	
Other	102 (10.3)	3 (13.0)		28 (11.2)	4 (6.5)	
Unknown	285 (28.9)	8 (34.8)		62 (24.9)	20 (32.3)	
**Ethnicity (%)**						
Not Hispanic/Latino	479 (48.5)	6 (26.1)	0.51	138 (55.4)	26 (41.9)	0.16
Hispanic/Latino	173 (17.5)	9 (39.1)		39 (15.7)	13 (21.0)	
Unknown	335 (33.9)	8 (34.8)		72 (28.9)	23 (37.1)	

Documentation about the source of the blood for the cultures was limited and unclear in many cases, but at least 10% were catheter-drawn, and at least 40% of those did not have an additional percutaneous culture within ±48 h. After the first positive culture in confirmed cases, the median interval between subsequent cultures was 0.98 days IQR [0.62, 1.48]. Additionally, there were 389 CSF cultures drawn within 28 days of birth in this study cohort. Two neonates had positive CSF cultures, these are described below. Although most of the blood culture isolates were bacteria, there were six neonates that were infected with yeast. Three were infected with *Candida lusitaniae*, one with *Candida dubliniensis*, and the species of the remaining two could not be determined from the records.

Specific organisms associated with confirmed sepsis are outlined in **Table 2**. Blood cultures that were drawn within 28 days of birth in all sepsis cohorts numbered 2,483; within these, 223 (8.98%) contained organisms considered as pathogens and 118 (4.75%) cultures were identified as possibly contaminated. Of the cultures that were possibly contaminated, 21 (17.8%) did not have a second blood culture draw within ±48 h. Of the organisms identified as possible contaminants, only coagulase-negative staphylococci (CoNS) was also identified as a potential pathogen. Out of 226 cultures that were positive for CoNS, 122 (54.0%) were categorized as pathogenic.

**Table 2:** Pathogens identified in culture-confirmed sepsis.

Organism	Early-onset sepsis* (%) n = 24	Late Onset sepsis* (%) n = 68
**Gram-positive bacteria**		
CoNS	1 (4.2)	31 (45.6)
Enterococcus spp.	1 (4.2)	7 (10.3)
*Staphylococcus aureus*		7 (10.3)
Group B *Streptococcus*	4 (16.7)	N/D
*Listeria monocytogenes*	1 (4.2)	N/D
*Streptococcus pneumoniae*	1 (4.2)	N/D
**Gram-negative bacteria**		
*Escherichia coli*	10 (41.7)	9 (13.2)
*Klebsiella pneumoniae*	2 (8.3)	3 (4.4)
Enterobacter spp.	N/D	2 (2.9)
*Serratia marcescens*	N/D	2 (2.9)
*Haemophilus influenzae*	2 (8.3)	N/D
*Acinetobacter* sp.	N/D	1 (1.5)
*Citrobacter sedlakii*	N/D	1 (1.5)
*Neisseria meningitidis*	1 (4.2)	N/D
Yeast	1 (4.2)	5 (7.4)

*Number of unique patient-organism combinations. (A patient may have cultured more than one organism.); N/D, not detected.

### Early-Onset Sepsis

There were 1,010 neonates within the EOS cohorts, 23 (2.3%) were classified as confirmed cases of EOS and 987 (97.7%) as unconfirmed (**Figure 1**). There were 24 culture isolates identified that were associated with EOS (**Table 2**); 1 of 23 (4.3%) neonates with confirmed EOS had more than one type of non-contaminant organism (*Escherichia coli* with yeast). The most common organisms identified as EOS pathogens were *Escherichia coli* (43.5% of neonates) and group B streptococcus (17.4%). One confirmed EOS patient infected with *Escherichia coli* had both blood and CSF cultures positive for *Escherichia coli*.

The ratio of culture confirmed to unconfirmed EOS cases remained consistent over time (p = 0.59). Because of the low number of confirmed EOS cases and the consistent occurrence, trends over time were analyzed for the combined confirmed and unconfirmed EOS cohorts. There was no statistically significant change in severity of illness and risk of mortality over time. The average gestational age and birth weight for EOS neonates increased over the study period from 30.4 to 34.5 weeks and 1.50 to 2.45 kg respectively (R^2^ = 0.05, p < 0.01 for both). The average length of stay for EOS neonates decreased from of 45.9 days at the beginning of the study period to 32.8 days at the end (R^2^ = 0.05, p < 0.01). Logistic regression showed that there was a statistically significant increase in the rate of mortality over time for EOS neonates (odds ratio 1.27/year, 95%CI [1.10, 1.49], p < 0.01).

**Figure 2:**
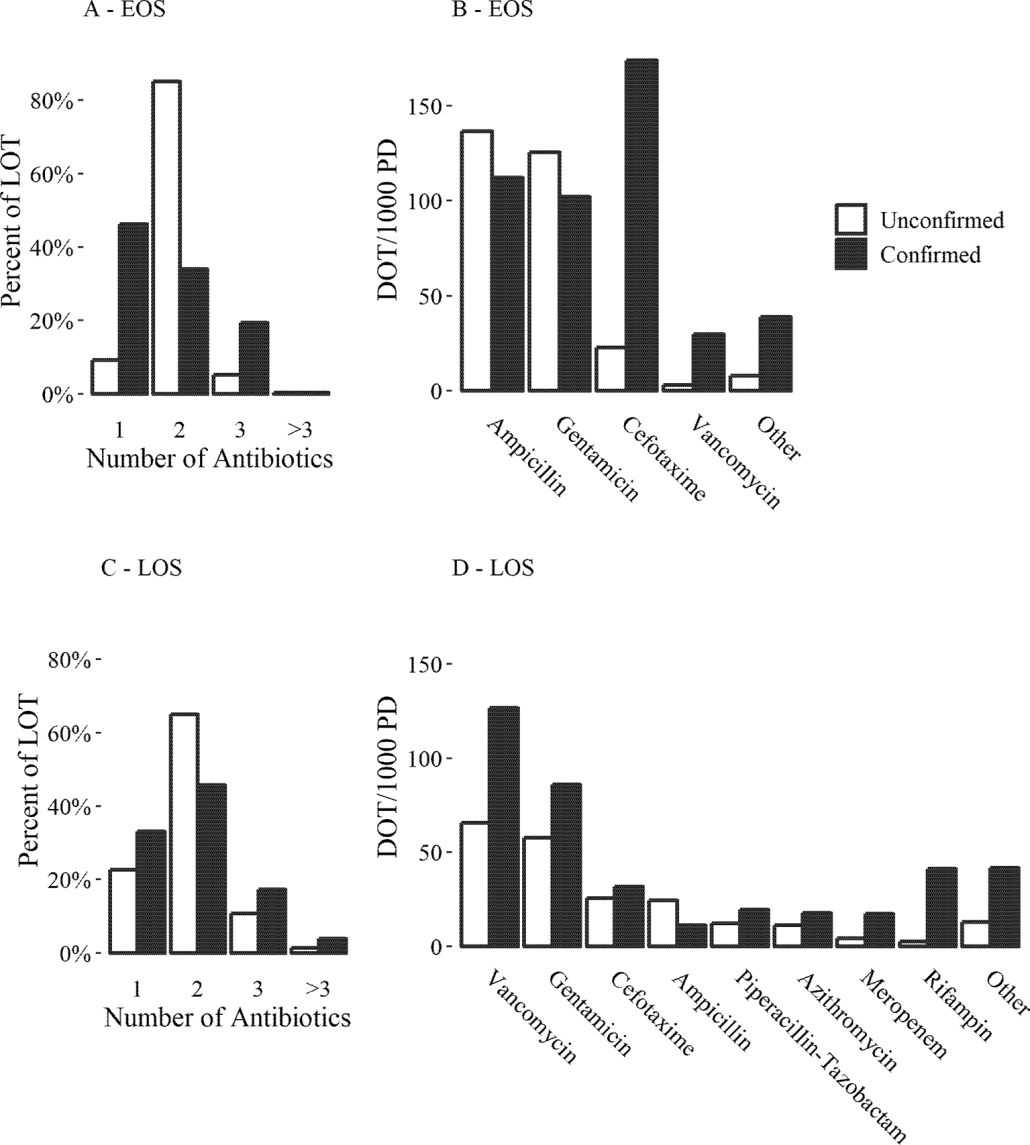
Antibiotic use for treatment of neonatal sepsis. **(A** and **C)** number of antibiotics administered per calendar day as a percentage of LOT per cohort. **(B** and **D)**—amount of each antibiotic administered as DOT/1,000 PD per cohort. **(A** and **B)** figures for early onset sepsis (EOS): only courses started within 3 days of birth are included. **(C** and **D)** figures for late onset sepsis (LOS): only courses started within >3–28 of birth were included.

Confirmed EOS neonates had more exposure to antibiotics than unconfirmed EOS neonates by all metrics in **Table 3** (p < 0.01). Unconfirmed EOS neonates were mostly prescribed ampicillin and gentamicin (**Figure 2B**); 77.2% of the LOT for courses starting within 3 days of birth in these patients included both ampicillin and gentamicin in combination. Confirmed EOS neonates were treated with only one antibiotic more often than unconfirmed EOS neonates (46.3% and 9.3% of LOT respectively, **Figure 2A**). For confirmed EOS neonates, 65.3% of the LOT for courses started within 3 days of birth included cefotaxime either alone or in combination with other antibiotics, 35.1% consisted of treatment with only cefotaxime. Cefotaxime was most common antibiotic for confirmed EOS neonates (**Figure 2B**). Fifteen of the confirmed EOS neonates received cefotaxime starting within 3 days of birth for a mean of 11.8 days LOT, SD (6.0). Gram negative microorganisms were cultured in 13 of the 15 confirmed EOS patients treated with cefotaxime and two of the patients died during the course of treatment.

**Table 3:** Measurements of total antibiotic exposure per cohort.

Metric	Early-Onset Sepsis*		Late-Onset Sepsis*	
	Suspected	Confirmed	Suspected	Confirmed
DOT/1000 PD (SD)	513 (477)	582 (334)	472 (314)	634 (385)
LOT/Admission (SD)	10.9 (10.0)	15.9 (7.0)	17.9 (11.5)	23.5 (14.9)
LOT/Course (starting ≤3 days of birth) (SD)	6.1 (3.4)	12.3 (6.2)	N/A	N/A
LOT/Course (starting >3–28 days of birth) (SD)	N/A	N/A	7.8 (5.7)	11.4 (9.4)

*The differences between suspected and confirmed sepsis were statistically significant for all metrics (p < 0.01).

There was no statistically significant changes in EOS course LOT over the study period for both unconfirmed and confirmed EOS (p = 0.45, 0.24 respectively). There was no significant change in the rates of administration of any antibiotic for confirmed EOS. Unconfirmed EOS courses saw a reduction in the rate of gentamicin administration and an increase in the rate of cefotaxime administration where very few courses included cefotaxime in at the beginning of the study period, to nearly 30% of courses including cefotaxime at the end of 2017 (**Table 4**).

**Table 4:** Antibiotics with significant changes in administration rates over time. Results of logistic regression modeling with antibiotic administration rates at the beginning and end of the time period (1/1/2006 - 12/31/2017).

Antibiotic	Odds ratio/year (95% CI)	P	Courses with Administration	
			Beginning	End
**Unconfirmed Early Onset Sepsis**				
Cefotaxime	1.25 (1.17, 1.34)	<0.01	2.8%	29.7%
Gentamicin	0.69 (0.57, 0.80)	<0.01	99.8%	87.2%
**Unconfirmed Late Onset Sepsis**				
Ampicillin	1.10 (1.02, 1.19)	0.02	28.9%	55.6%
Cefotaxime	1.24 (1.14, 1.37)	<0.01	8.4%	55.8%
Gentamicin	0.78 (0.70, 0.85)	<0.01	92.4%	36.3%
Piperacillin-Tazobactam	1.24 (1.12, 1.39)	<0.01	4.6%	39.8%
Vancomycin	0.83 (0.75, 0.89)	<0.01	86.8%	38.7%
**Confirmed Late Onset Sepsis**				
Gentamicin	0.78 (0.65, 0.91)	<0.01	94.0%	42.8%
Rifampin	0.79 (0.65, 0.93)	<0.01	58.3%	7.1%

### Late-Onset Sepsis

Of 311 neonates within the LOS cohorts, 62 (19.9%) were classified as confirmed cases of LOS, 249 (80.1%) as unconfirmed. A total of 68 culture isolates were identified as pathogens associated with LOS (**Table 2**); 4 of 62 (6.5%) confirmed LOS neonates were positive for multiple organisms (fist patient—CoNS, *Klebseilla pneumonia*, *Entercoccus faecalis*, and *Candida lusitaniae*; second—*Staphylococcus aureus* and *Enterococcus faecalis*; third—CoNS and *Acinetobacter* sp.; fourth—*Staphylococcus aureus and Staphylococcus epidermidis*). The most common pathogens causing LOS were CoNS (50.0% of neonates) followed by *Escherichia coli* (14.5%). One of the unconfirmed LOS neonates had one positive CSF culture within >3–28 days of birth which isolated *Candida dubliniensis*, no blood cultures were drawn within ±4 days of this CSF culture.

Similar to confirmation rates in EOS, the ratio of culture confirmed to unconfirmed LOS cases also remained consistent over time (p = 0.80). There was no statistically significant change in severity of illness, risk of mortality, gestational age, birth weight, and length of stay over time when unconfirmed and confirmed LOS were analyzed both combined and separated. Mortality rates within 35 days of birth increased over time for unconfirmed LOS (odds ratio 1.37/year, 95%CI [1.10, 1.81], p = 0.01) and confirmed LOS (odds ratio 1.42/year, 95%CI [1.06, 2.17], p = 0.04).

The most common antibiotics used in neonates with unconfirmed and confirmed LOS were vancomycin and gentamicin (**Figures 2C–D**). Similar to neonates with EOS, confirmed LOS neonates had more exposure to antibiotics than unconfirmed LOS by all metrics (**Figure 2C**, **Table 3**, p < 0.01). For courses starting within >3–28 days of birth, rifampin was more frequently administered to confirmed LOS neonates than unconfirmed (41.2 and 2.6 DOT/1000 PD respectively, **Figure 2D**). In all 22 cases of confirmed LOS treated with rifampin, the neonates were infected with CoNS and treatment was always combined with vancomycin. The mean time difference between the first positive culture draw and the start of rifampin treatment was 4.0 days SD (1.8). In 17 neonates (77%), there were no positive cultures after initiation of rifampin treatment, for the remaining neonates the mean time to the last positive culture after treatment was started was 1.85 days SD (1.92). One patient died after 2.5 days of treatment with rifampin; this patient had a positive culture drawn just prior to death. There was no statistically significant changes in LOS course LOT over the study period for both unconfirmed and confirmed LOS (p = 0.93, 0.57 respectively). There was a reduction in the rate of rifampin administration in cases of confirmed LOS, a reduction in vancomycin administration for unconfirmed LOS, and a reduction in gentamicin administration over time in both cohorts (**Table 4**). Courses for unconfirmed LOS saw an increase in the inclusion of ampicillin, piperacillin-tazobactam, and cefotaxime (**Table 4**).

### Neonatal Mortality

As described, patient mortality had statistically significant increasing trend over the study period for unconfirmed EOS and LOS and confirmed LOS. For the combined EOS and LOS cohorts, a logistic regression model estimated a mortality rate within 35 days of birth of 0.4% at the beginning of the study period to 8.5% of patients at the end (odds ratio 1.30/year, 95%CI [1.13, 1.52], p < 0.01). Additionally, the use of cefotaxime was included in treatment courses for unconfirmed EOS and LOS at an increasing rate during the study period (**Table 4**). For combined EOS and LOS neonates, the inclusion of cefotaxime within 28 days of birth went from 5.6% of courses to 37.6% of courses (odds ratio 1.21, 95%CI [1.16, 1.27], p < 0.01) over the course of the study period.

When mortality was modeled as a function of whether neonates had exposure to cefotaxime within 28 days of birth, cefotaxime exposure had a significant impact on mortality (odds ratio 7.92, 95%CI [3.76, 17.79], p < 0.01). This association remained when gestational age, birth weight, hospital admission date-time, blood culture confirmation of sepsis, risk of mortality, and severity of illness were added to the logistic regression model as potential confounders (adjusted odds ratio 2.8, 95%CI [1.21, 6.88], p = 0.02).

## Discussion

### Confirmation of Neonatal Sepsis

Establishing blood culture-confirmed neonatal sepsis is complicated by the presence of false negative culture results and culture contamination. False negative cultures are commonly attributed to low volume of blood or low pathogen concentrations, which is an inherent problem in the mostly low body weight newborns with neonatal sepsis (Kellogg et al., 2000; Dien Bard and McElvania TeKippe, 2016). Additionally, it is believed by some that exposure to intrapartum antibiotics when treating chorioamnionitis or other maternal illnesses can reduce sensitivity of blood cultures (Peralta-Carcelen et al., 1996). To overcome these limitations, it is recommended that ≥ ≥1 ml of blood is collected for culturing (Polin, 2012; Dien Bard and McElvania TeKippe, 2016). Using 1 ml of blood for a blood culture has been demonstrated to provide 98% sensitivity even when pathogen concentrations are low (4 colony forming units per ml) (Schelonka et al., 1996). The volume of cultured blood was not available in this study, therefore the effect of blood volume on false negative results in this hospital system are difficult to infer.

Even though a blood culture may provide a sensitive diagnostic tool, the results may take several days. In the past, attempts have been made to detect true cases of sepsis earlier than a blood culture, (Philip and Hewitt, 1980) but there is no consensus on how to incorporate laboratory tests in diagnosing neonatal sepsis (Wynn et al., 2014). New biomarkers such as bacterial surface antigens and emerging technologies such as genetic sequencing based tools may be used more frequently in neonatal sepsis diagnostics in the future (Chauhan et al., 2017; Iroh Tam and Bendel, 2017). Automated multiplex PCR systems can provide pathogen specific identification of common microorganisms including many organisms found in neonates such as *E.coli* and *S. aureus* in as little as an hour, (Salimnia et al., 2016) but it is unknown whether all pathogens are adequately detectable.

Regardless of the method used to detect microorganisms in a blood sample, sample contamination can mislead diagnosis. To complicate the issue, when a central venous catheter is used to draw blood, positive cultures may be due to catheter colonization (Hall and Lyman, 2006). Some organisms, such as *Escherichia coli*, *Staphylococcus aureus*, and *Streptococcus pneumoniae*, are almost always associated with a true infection, while others are commonly contaminants, such as CoNS and *Streptococcus viridans* (Hall and Lyman, 2006). When a common contaminant is cultured, misdiagnosis is reduced by obtaining and comparing multiple cultures; and when blood is drawn from a catheter, a second sample should be drawn percutaneously (Schifman et al., 1998; Hall and Lyman, 2006). CoNS are the most common blood culture contaminants, (Hall and Lyman, 2006) but they are also a prominent causative agent of LOS (Dong and Speer, 2015). In this study, approximately 54% of cultures positive for CoNS were identified as true positives, 46% were identified as potentially contaminated. Additionally, at least 15% of CoNS-positive cultures were from catheter-drawn blood samples. Distinguishing between contaminants and pathogens is imperative in these cases. CoNS cultures cannot simply be completely excluded from, or completely included in, culture-confirmed cases.

Clinical signs and laboratory results indicating sepsis beyond blood cultures are commonly used to classify confirmed cases of sepsis (Bizzarro et al., 2005; Haque, 2005; Wynn et al., 2014; Zea-Vera and Ochoa, 2015). Incorporating any of these methods into confirming sepsis retrospectively has shortcomings; in particular, data are sometimes sparse. It is important to realize that when laboratory results or clinical signs remain abnormal but blood cultures are negative, sepsis may still be suspected and the use of antibiotics may be prolonged. To allow comparisons with other studies and since blood cultures are the gold standard for the diagnosis of neonatal sepsis, the criteria for retrospectively confirming neonatal sepsis *via* a modified version of CDC guidelines (Horan et al., 2008) within this study used only culture results.

### Treatments for Neonatal Sepsis

Of neonates treated for EOS in the University of Utah Hospital system, only 2.3% had blood culture confirmation and there was a high rate of empiric use of ampicillin and gentamicin. This type of empiric treatment is common practice in neonatal intensive care units (NICUs) within the United States. Oliver et al. reported that within the Pediatric Health Information System, 92.5% of neonates in a NICU that were administered antibiotics within three days of birth received ampicillin and/or gentamicin on the first day of treatment (Oliver et al., 2017). Cefotaxime appeared to be a preferred choice in therapy for confirmed EOS with gram-negative infections for the University of Utah Hospital system.

Empiric treatment for LOS seems to vary more than empiric treatment for EOS, but within the University of Utah Hospital system, vancomycin and/or gentamicin were the most common treatments. In a 2002 survey of 278 neonatology clinicians at 35 hospitals with NICUs, at least 60% prescribed vancomycin as part of empiric therapy for unconfirmed LOS, commonly combined with an aminoglycoside (Rubin et al., 2002). Prudent use of vancomycin has been advised for decades due to the evolution of vancomycin resistant strains of pathogens (The Hospital Infection Control Practices Advisory Committee, 1995). There is some evidence that empiric vancomycin does not improve short-term mortality or length of stay when neonates are infected with CoNS (Ericson et al., 2015). Within the University of Utah Hospital system, there are signs of improvement in the antibiotic stewardship of vancomycin where the DOT/1,000 PD for vancomycin use was 37.3% lower in unconfirmed compared to confirmed cases of LOS with a significant reduction in empiric use over time (**Table 4**). Rifampin was often combined with vancomycin to treat persistent CoNS infections within the University of Utah Hospital system—there is some limited evidence that this strategy is effective (Acar et al., 1983; Shama et al., 2002; van der Lugt et al., 2010; Rodriguez-Guerineau et al., 2013).

Neonates with unconfirmed sepsis within the University of Utah Hospital system were treated for a median of 7 days LOT IQR (Clark et al., 2006; Liu et al., 2012), which is similar to, or longer than, treatment lengths for unconfirmed sepsis in similar studies within the United States and Europe (Patel et al., 2009; Cantey et al., 2015; Fjalstad et al., 2016). Antibiotic administration may be prolonged even when cultures are negative if blood cell counts, C reactive protein levels, or other diagnostic tests remain abnormal. Continued administration of antibiotics when cultures are negative appears to be a common practice. This is in spite of evidence that prolonged empirical antibiotic administration within neonates (≥5 days) is associated with adverse outcomes such as necrotizing enterocolitis, prolonged hospital stay, and death (Kuppala et al., 2011; Ting et al., 2016). Recent studies on the incubation time for blood cultures in cases of neonatal sepsis suggest that antibiotic treatment can be ceased after 48 h if cultures remain negative (Jardine et al., 2006; Durrani et al., 2019) Cantey et al. demonstrated that when empiric neonatal antibiotic therapy was set to automatically discontinue after 48 h and continued therapy was limited to 5 days when cultures were negative, there was no difference in safety outcomes compared to prior practices where antibiotics were regularly administered ≥6 days when cultures were negative (Cantey et al., 2016).

Since the gestational age, birth weight, severity of illness, risk of mortality, and the ratio of culture confirmed to unconfirmed cases of neonatal sepsis remained fairly consistent throughout the study period, it was unexpected that the neonatal sepsis mortality rate within the University of Utah Hospital system increased over the study period. In 2006, Clark et al. showed that the concurrent use of ampicillin and cefotaxime within 3 days of birth rather than ampicillin with gentamicin had an increased risk of mortality (Clark et al., 2006). Logistic modeling showed that the use of cefotaxime within 28 days of birth may be a cause of the increased neonatal sepsis mortality within the University of Utah hospital system, even after adjusting for potential confounders. This conclusion is limited by the retrospective nature of this study: cefotaxime administration may be a surrogate for some other unknown factor. Further analysis of the causes of death and the motivations for prescribing cefotaxime may reveal other factors that may be responsible for the increased mortality rate.

One potential limitation of this study is that antibiotic therapy may have been prolonged for neonates with infections other than sepsis such as pneumonia or meningitis. In our patient cohorts, there were no neonates that had both negative blood cultures and positive CSF culture which would suggest neonatal meningitis. When neonates with any other positive cultures were removed from the unconfirmed cohorts (see **Table 1**), the LOT/course for courses starting ≤3 days of birth reduced from 6.1 to 6.0 for unconfirmed EOS and the LOT/course for courses starting >3–28 days of birth reduced from 7.8 to 7.5 for unconfirmed LOS. Both of these differences were insignificant (p > 0.05); therefore, we believe that the main findings of this study are still supported. This study has other limitations. Comorbidities and mother’s characteristics were not accounted for and may affect suspicion of sepsis and choices in antibiotic treatments. The results of this study cannot be generalized to other hospitals since epidemiology and practices are likely to differ from region to region.

## Twelve-Year Overview

Over the past 12 years, the level of empirical treatment remained high. The major limitations to reduce empirical treatment are highly variable clinical presentation of sepsis in neonates and low levels of confirmed sepsis. The development or improvement of diagnostic tests capable of accurately confirming sepsis within 1–2 h after blood draw is a key factor that will reduce empirical treatment of neonatal sepsis. One of the possible risk factors for the development of sepsis in neonates is an underdeveloped immune defences (Gervassi and Horton, 2014). Recently, it has been proposed that immune responses in neonates are suppressed by afterbirth residual myeloid derived suppressor cells (MDSCs) (Gervassi and Horton, 2014). MDSCs are a heterogeneous population of immature myeloid cells capable of expanding following pathologic conditions such as infections and cancer. MDSCs have been detected in the blood of septic patients (Cuenca et al., 2011). The ontogenetic destiny of MDSCs is to suppress the fetal immune response against the mother. MDSCs possess the remarkable ability to suppress T, B, and NK cell functions and by this mean impair protective immune responses to infectious agents (Rieber et al., 2013; Gervassi et al., 2014). Presence of these cells in cord blood can potentially serve as early biomarker of neonatal sepsis. If MDSCs indeed suppress anti-microbial immunity of neonates, the immune system can be restored and sepsis development prevented by blocking MDSCs activity or by differentiation of MDSCs into mature myeloid cells lacking suppressive activities. This approach may reduce the rate of neonatal sepsis, empirical use of antibiotics, and increase survival of preterm babies.

## Conclusions

In conclusion, the large proportion of unconfirmed to confirmed neonatal sepsis combined with a high empiric antibiotic administration rate and high level of exposure indicates an opportunity to reduce unnecessary antibiotic use in the neonatal population. This may be accomplished by implementing new and improved methods for more sensitive and timely diagnosis of neonatal sepsis and reevaluating guidelines for appropriate antibiotic use.

## Data Availability Statement

The dataset for this manuscript is not publicly available because it contains personal information on human subjects. The dataset cannot be completely de-identified and shared without compromising the privacy of research participants.

## Ethics Statement

The studies involving human participants were reviewed and approved by University of Utah Institutional Review Board (IRB_00091312). Written informed consent from the participants’ legal guardian/next of kin was not required to participate in this study in accordance with the national legislation and the institutional requirements.

## Author Contributions

JW, MN and RD undertook the analysis, EE and CS designed and conceptualized the study, EE, CS, RE and RW were involved in study design and data analysis. All authors were engaged in the writing and review of the manuscript drafts and final submission.

## Conflict of Interest

CS is the Specialty Chief Editor of the section Obstetric and Pediatric Pharmacology within Frontiers in Pharmacology.

The remaining authors declare that the research was conducted in the absence of any commercial or financial relationships that could be construed as a potential conflict of interest.
